# Protective role of SARS-CoV-2 anti-S IgG against breakthrough infections among European healthcare workers during pre and post-Omicron surge—ORCHESTRA project

**DOI:** 10.1007/s15010-024-02189-x

**Published:** 2024-02-07

**Authors:** Gianluca Spiteri, Marika D’Agostini, Mahsa Abedini, Giorgia Ditano, Giulia Collatuzzo, Paolo Boffetta, Luigi Vimercati, Emanuele Sansone, Giuseppe De Palma, Alberto Modenese, Fabriziomaria Gobba, Filippo Liviero, Angelo Moretto, Marco dell’Omo, Tiziana Fiordi, Francesca Larese Filon, Marcella Mauro, Concepción Violán, Dana Mates, Jana Oravec Bérešová, Maria Grazia Lourdes Monaco, Angela Carta, Giuseppe Verlato, Stefano Porru

**Affiliations:** 1 Occupational Medicine Unit, University Hospital of Verona, 37134 Verona, Italy; 2 Department of Medical and Surgical Sciences, University of Bologna, 40138 Bologna, Italy; 3 Interdisciplinary Department of Medicine, University of Bari, 70121 Bari, Italy; 4 Unit of Occupational Health and Industrial Hygiene, Department of Medical and Surgical Specialties, Radiological Sciences and Public Health, University of Brescia, 25121 Brescia, Italy; 5 Unit of Occupational Health, Hygiene, Toxicology and Prevention, University Hospital ASST Spedali Civili, 25123 Brescia, Italy; 6 Department of Biomedical, Metabolic and Neural Sciences, University of Modena and Reggio Emilia, 41125 Modena, Italy; 7 Department of Cardiac Thoracic Vascular Sciences and Public Health, University of Padova, 35128 Padua, Italy; 8 Occupational Medicine Unit, University Hospital of Padova, 35128 Padua, Italy; 9 Department of Medicine and Surgery, University of Perugia, 06129 Perugia, Italy; 10 Occupational Medicine Unit, Perugia Hospital, 06129 Perugia, Italy; 11 Department of Medical Sciences, Unit of Occupational Medicine, University of Trieste, 34129 Trieste, Italy; 12 Unitat de Suport a la Recerca Metropolitana Nord, Institut Universitari d’Investigació en Atenció Primària Jordi Gol (IDIAP Jordi Gol), 08303 Mataró, Barcelona, Spain; 13 Germans Trias i Pujol Research Institute (IGTP), 08916 Badalona, Spain; 14 Grup de REcerca en Impacte de les Malalties Cròniques i les Seves Trajectòries (GRIMTra), (2021 SGR 01537), Institut Universitari d’Investigació en Atenció Primària Jordi Gol (IDIAPJGol), 08303 Barcelona, Spain; 15 Network for Research on Chronicity, Primary Care, and Health Promotion (RICAPPS) (RD21/0016/0029), Insitituto de Salud Carlos III, 28029 Madrid, Spain; 16 Direcció d’Atenció Primària Metropolitana Nord Institut Català de Salut, 08204 Barcelona, Spain; 17 Universitat Autónoma de Barcelona, 08193 Bellaterra, Spain; 18 National Institute of Public Health, 050463 Bucharest, Romania; 19 Epidemiology Department, Regional Authority of Public Health, 97556 Banská Bystrica, Slovakia; 20 Section of Occupational Medicine, Department of Diagnostics and Public Health, University of Verona, 37134 Verona, Italy; 21 Unit of Epidemiology and Medical Statistics, Department of Diagnostics and Public Health, University of Verona, 37134 Verona, Italy

**Keywords:** Healthcare workers, COVID-19, SARS-CoV-2, SARS-CoV-2 anti-S IgG, COVID-19 vaccination, Breakthrough infections, Omicron variant

## Abstract

**Purpose:**

Anti SARS-CoV-2 vaccination initially showed high effectiveness in preventing COVID-19. However, after the surge of variants of concern, the effectiveness dropped. Several studies investigated if this was related to the decrease of the humoral response over time; however, this issue is still unclear. The aim of this study was to understand whether SARS-CoV-2 anti-S IgG levels can be used to predict breakthrough infection risk and define the timing for further booster doses administration.

**Method:**

Within the framework of the ORCHESTRA Project, over 20,000 health workers from 11 European centers were enrolled since December 2020. We performed two Cox proportional hazards survival analyses regarding pre-Omicron (from January to July 2021) and Omicron (December 2021–May 2022) periods. The serological response was classified as high (above the 75th percentile), medium (25th-75th), or low (< 25th).

**Results:**

Seventy-four (0.33%) and 2122 (20%) health workers were infected during the first and second periods, respectively. Both Cox analyses showed that having high anti-S titer was linked to a significantly lower risk of infection as compared to having medium serological response [HR of high vs medium anti-S titer = 0.27 (95% CI 0.11–0.66) during the first phase, HR = 0.76 (95% CI 0.62–0.93) during the second phase].

**Conclusion:**

Vaccine effectiveness wanes significantly after new variants surge, making anti-S titer unsuitable to predict optimal timing for further booster dose administration. Studies on other immunological indicators, such as cellular immunity, are therefore needed to better understand the mechanisms and duration of protection against breakthrough infection risk.

**Supplementary Information:**

The online version contains supplementary material available at 10.1007/s15010-024-02189-x.

## Introduction

In November 2019, a new coronavirus was detected in China. After the rapid spread worldwide, WHO classified the SARS-CoV-2 infection as a global pandemic on 11 March 2020, characterized by high mortality among vulnerable categories. As a consequence, enormous efforts have been made to produce an effective vaccine against the virus, and, starting from December 2020, the vaccine administration began in the European population, prioritizing the high-risk groups, including the elderly and the health workers (HW). General population’s phase 3 trials and observational studies among HW showed very high effectiveness against SARS-CoV-2 infection and COVID-19 severity during the first few months after a full vaccination course [1–4]. However, since the onset and the spread of variants of concern (VOC), protection against infection diminished significantly. In particular, after the emergence of the Omicron variant (OV) in November 2021, and the subsequent increase in the number of new cases, the WHO recommended the administration of a booster dose, with the aim of restoring the immune response and reducing the risk of infection. A previous study involving the ORCHESTRA project Cohorts, showed that this intervention reduced the chance of being infected and the COVID-19 severity during the OV period, although the effectiveness was significantly reduced when compared to previous variants [5]. A significant reduction in symptoms after OV SARS-CoV-2 infection among boostered HW towards not-boostered ones was also reported in a study carried out by Kohler et al. [6]. To understand the role of humoral response in the waning of vaccine protection, several studies investigated the capacity of vaccines to trigger anti-S antibody production among general and HW populations and the correlation between the antibody titer and the risk of breakthrough infection (BI) [7–10]. However, this issue remains unclear. Indeed, although more than 99% of the HW belonging to the ORCHESTRA project developed an antibody titer above the positivity threshold, remaining positive 6 and 9 months after the second dose [11–13], they still had BI during the period of the pre-Omicron variants (POV), and even more during the OV, despite the effect of the third dose on antibody titer [1, 5, 14].

The aims of this study were to understand whether, based on data derived from a large population of HW belonging to different European cohorts, the assessment of SARS-CoV-2 anti-S IgG levels can be used as a predictor of:

- BI risk during different VOC periods.
- timing for further booster doses administration.

## Methods

### Study design and setting

We used data from a multicentre retrospective cohort study of HW (including physicians and nurses, as well as technicians, other HW, and administrative workers) within the Horizon 2020 ORCHESTRA research project [15].

Data were collected from different centres in Italy (Bari, Bologna, Brescia, Modena, Padova, Perugia, Trieste, and Verona), Spain (Northern Barcelona region), Romania, and Slovakia.

Participants were enrolled from 27 December 2020. The follow-up ended on 31 May 2022. Data on sociodemographic characteristics such as sex, age, and job title, as well as COVID-19 PCR testing and vaccination status (including date of vaccination, number of doses, and type of vaccine administered) were obtained from medical surveillance records, ad-hoc questionnaires and/or ongoing loco-regional databases. All data were collected in a pseudoanonymized format, managed using the REDCap electronic data capture tools hosted at the Italian Interuniversity Consortium CINECA and undergone extensive data harmonization.

This study followed the “Strengthening the Reporting of Observational Studies in Epidemiology” (STROBE) reporting guidelines and was approved by the Italian Medicine Agency (AIFA) and the Ethics Committee of the Italian National Institute of Infectious Diseases (INMI) Lazzaro Spallanzani. The study was also approved by the local ethical boards.

### Case definition and inclusion criteria

A participant was categorized as infected if tested positive on at least one nasopharyngeal swab during the study period Only HW who performed one or more serological tests were involved in the analysis. For not infected HW, we considered the latest sample performed during the timeframe, while for the infected ones, the sample closest to the date of the PCR positivity. Only serology tests performed at least 14 days before the infection were evaluated.

Only HW who completed the first vaccination course were included in the he first analysis, focused on the POV (Phase 1 analysis, from January 2021 to July 2021).

The second analysis, focused on OV period (December 2021–May 2022), included only HW who were vaccinated with a booster dose.

Details on the HW selection process are presented in Fig. S1and S2.

### Statistical analysis

Continuous variables were summarized using median with InterQuartile Range (IQR) whereas categorical variables were described by frequencies, and percentages.

Methods of measurement of the antibody levels varied between the included centres and in the time periods, therefore different analytical methods were used for different cohorts. Due to the different limits of detection presented in the serology test samples, cohort-specific Tobit regression models have been used to predict right-censored serology measurements in Brescia, Bologna, and Perugia cohorts, and to predict both left and right-censored serology measurements in Slovakia cohort. Information on sex, age, number of COVID-19 vaccine doses, and previous COVID-19 infection were used as predictive variables in the Tobit regression models. We then log-transformed the results of all the cohorts included in the study and divided them by the cohort-specific standard deviation (SD). Standardized serological measurements were obtained, allowing comparison across cohorts within the study population. The same approach was used in previous analyses within ORCHESTRA Project [11, 13, 14].

Two multiple-record stratified time-to-event analyses (also called survival analyses) using the technique of Cox Proportional Hazards (PH) regression were performed afterwards, one focusing on the POV period (Phase 1 analysis) and one focusing on the times of OV (Phase 2 analysis). The date of SARS CoV-2 infection is set to be the failure event of the survival models and the time variable is calculated from the date of the serology measurement. The exit time corresponds to when the failure event occurs or, in the case of not infected HW, to the end of the model-specific timeframe.

Cohorts to which HW belong and the number of days between the last vaccine dose and the time of the serology measurement (< 60 days; 60–89 days; ≥ 90 days for the first model, and < 60 days; 60–89 days; 90–119 days; ≥ 120 days for the second one) have been used as stratified variables. Sex, age group (categories: 18–29; 30–39; 40–49; 50–59; ≥ 60), job title (Physicians, Nurses, Technicians, Administratives, Other HW), previous infections (No previous infection; Before 1st vaccine dose; After 1st vaccine dose) and the degree of serological response (categories: High, above or equal to the 75th percentile of the overall standardized serology results distribution; Low, below or equal to the 25th percentile of the overall standardized serology results distribution; Medium, between the 25th and 75th percentile) have been used as model covariates. Other variables such as the type of vaccination administrated were not included in the model since they were redundant and/or not informative. Robust variance estimators for the variance–covariance matrix of the coefficients (and hence the reported standard errors) were adopted to adjust for the clustered structure of data.

The Kaplan–Meier (KM) estimate [16] was used to graphically compare survival over time and the equality of survival functions was tested using the log-rank test. PH assumption was tested based on Schoenfeld residuals. Survival model results are expressed as hazard ratio (HR) and corresponding 95% confidence intervals (CI).

Statistical analysis was performed using Stata^®^ software 17.0 (StataCorp. 2021. Stata Statistical Software: Release 17. College Station, TX: StataCorp LLC).

## Results

### Description of participants

A total of 22,293 and 10,612 HW were included in the first analysis focused on the POV period (Phase 1 analysis) and in the second analysis on the OV (Phase 2 analysis), respectively. The distribution of HW within the different cohorts is shown in Table 1.

**Table 1:** Distribution of the health workers included in pre-Omicron and Omicron periods analyses, by Cohort

Cohort	Pre-Omicron period			Omicron period		
	Total (*N* = 22,293) frequency (%)	Not infected HW (*N* = 22,219) frequency (%)	Infected HW (*N* = 74) frequency (%)	Total (*N* = 10,612) frequency (%)	Not infected HW (*N* = 8,490) frequency (%)	Infected HW (*N* = 2,122) frequency (%)
Italy—Bari	345 (1.5)	341 (1.5)	4 (5.4)	–	–	–
Italy—Bologna	6,503 (29.2)	6,488 (29.2)	15 (20.3)	743 (7.0)	650 (7.7)	93 (4.4)
Italy—Brescia	6,294 (28.2)	6,282 (28.3)	12 (16.2)	4,816 (45.4)	4,265 (50.2)	551 (26.0)
Italy—Modena	1,418 (6.4)	1,406 (6.3)	12 (16.2)	–	–	–
Italy—Padova	–	–	–	604 (5.7)	124 (1.5)	480 (22.6)
Italy—Perugia	1,781 (8.0)	1,780 (8.0)	1 (1.3)	250 (2.4)	247 (2.9)	3 (0.1)
Italy—Trieste	4,328 (19.4)	4,315 (19.4)	13 (17.6)	677 (6.4)	505 (5.9)	172 (8.1)
Italy—Verona	879 (4.0)	876 (4.0)	3 (4.1)	2,961 (27.9)	2,153 (25.4)	808 (38.1)
Romania	450 (1.0)	450 (1.0)	–	148 (1.4)	148 (1.7)	–
Slovakia	217 (2.0)	217 (2.0)	–	235 (2.2)	223 (2.6)	12 (0.6)
Spain—Barcelona	78 (0.3)	64 (0.3)	14 (18.9)	178 (1.7)	175 (2.1)	3 (0.1)

The median age of participants was 48 years (IQR 36–56) in Phase 1 analysis and 49 years (IQR 38–56) in Phase 2. The majority of participants were female in both periods (69% and 74%, respectively). Socio-demographic and laboratory characteristics of the study population are reported in Table 2

**Table 2:** Socio-demographic and laboratory characteristics of Health Workers included in the study

Variable	Pre-Omicron period				Omicron period			
	Total (*N* = 22,293) frequency (%)	Not infected HW (*N* = 22,219) frequency (%)	Infected HW (*N* = 74) frequency (%)	Stratified log-rank test, *P* value (*)	Total (*N* = 10,612) frequency (%)	Not infected HW (*N* = 8,490) frequency (%)	Infected HW (*N* = 2,122) frequency (%)	Stratified log-rank test, *P* value (*)
Sex								
Female	15,373 (69.0)	15,322 (69.0)	51 (69.0)	0.113	7,775 (73.3)	6,209 (73.1)	1,566 (73.8)	0.269
Male	6,920 (31.0)	6,897 (31.0)	23 (31.0)		2,837 (26.7)	2,281 (26.9)	556 (26.2)	
Age								
18–29	2,060 (9.2)	2,051 (9.2)	9 (12.2)	0.726	1,058 (10.0)	757 (8.9)	301 (14.2)	0.002
30–39	5,103 (22.9)	5,090 (22.9)	13 (17.6)		1,928 (18.2)	1,479 (17.4)	449 (21.2)	
40–49	4,967 (22.3)	4,944 (22.3)	23 (31.1)		2,473 (23.3)	1,933 (22.8)	540 (25.4)	
50–59	7,098 (31.8)	7,077 (31.8)	21 (28.3)		3,899 (36.7)	3,225 (38.0)	674 (31.8)	
≥ 60	3,065 (13.8)	3,057 (13.8)	8 (10.8)		1,254 (11.8)	1,096 (12.9)	158 (7.4)	
Job title								
Physicians	6,143 (27.6)	6,120 (27.5)	23 (31.1)	0.073	2,308 (21.8)	1,806 (21.2)	502 (23.7)	< 0.001
Nurses	8,180 (36.7)	8,144 (36.7)	36 (48.6)		4,025 (37.9)	3,080 (36.3)	945 (44.5)	
Technicians	2,274 (10.2)	2,271 (10.2)	3 (4.1)		1,108 (10.4)	915 (10.8)	193 (9.1)	
Administrative	1,610 (7.2)	1,607 (7.2)	3 (4.1)		1,096 (10.3)	949 (11.2)	147 (6.9)	
Other HW	4,086 (18.3)	4,077 (18.4)	9 (12.1)		2,075 (19.6)	1,740 (20.5)	335 (15.8)	
Previous infections								
No	22,288 (100)	22,219 (100)	69 (93.2)	< 0.001	9,754 (91.9)	7,809 (92.0)	1,945 (91.7)	0.204
Before 1st vaccine dose	4 (0.0)	–	4 (5.4)		810 (7.6)	681 (8.0)	129 (6.1)	
After 1st vaccine dose	1 (0.0)	–	1 (1.4)		48 (0.5)	–	48 (2.2)	
Serological response								
High	5,573 (25.0)	5,544 (24.9)	29 (39.2)	0.016	2,734 (25.8)	2,644 (31.1)	90 (4.2)	0.041
Medium	11,144 (50.0)	11,121 (50.1)	23 (31.1)		5,227 (49.2)	3,799 (44.8)	1,428 (67.3)	
Low	5,576 (25.0)	5,554 (25.0)	22 (29.7)		2,651 (25.0)	2,047 (24.1)	604 (28.5)	

(*) Log-rank to compare the survival distributions of the groups defined by the different values of the categorical variables

### COVID-19 infections and serology measurements

**Fig. 1:**
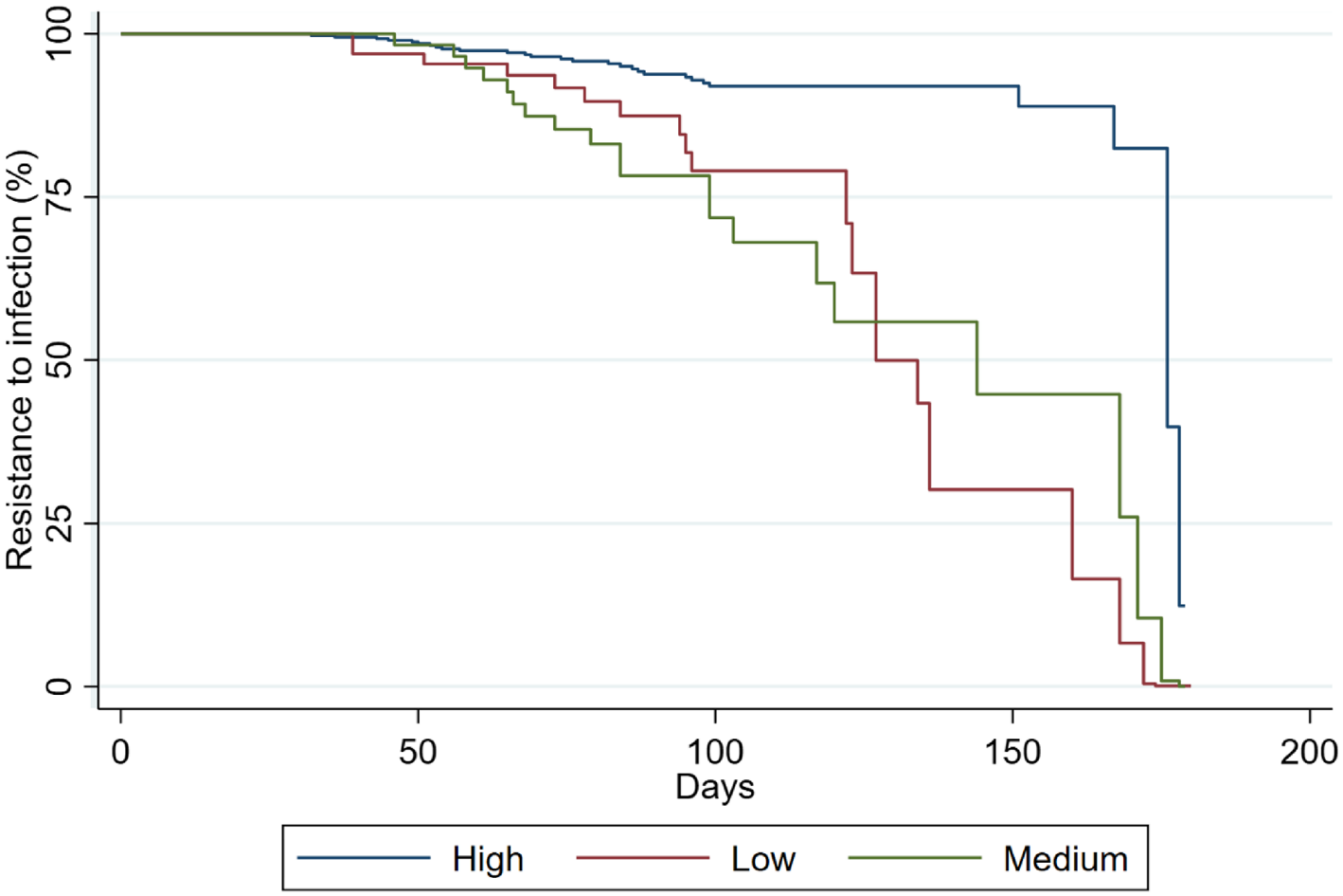
Kaplan–Meier survival curves by antibody levels among health workers involved in the pre-Omicron period analysis

A total of 74 (0,3%) HW were infected during Phase 1 analysis. The median time-to-event (where event should be understood as the SARS-CoV-2 infection) was 104 days (IQR 26–129). The survival function is shown in Fig. 1. The median number of days elapsed between the second vaccine dose and the serology test was 100 days (IQR 60–127). The median standardized serology measurement was 6.25 (IQR 5.48–7.18).

**Fig. 2:**
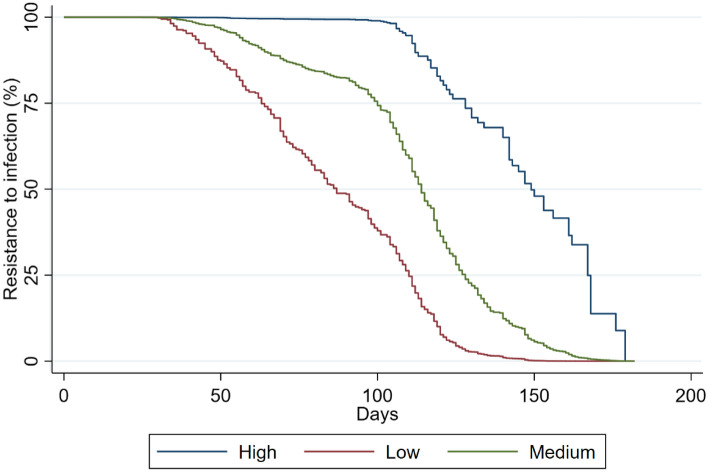
Kaplan–Meier survival curves by antibody levels among health workers involved in the Omicron period analysis

Regarding Phase 2 analysis, 2122 (20%) HW had SARS-CoV-2 infection. The median survival time was 85 days (IQR 43–94). The survival function is shown in Fig. 2. The median number of days after the full vaccination course completion and the serology test was 85 days (IQR 49–117). The median standardized serology measurement was 11.55 (IQR 10.21–12.42).

### Survival analyses and factors associated with the risk of COVID-19 infection

Analysing the KM survival curves related to antibody levels of HW involved in Phase 1 analysis (Fig. 1), we observed that HW with higher anti-S titers (blue curve) had higher disease-free survival probability throughout the period than HW who had a medium-level serological response (green curve).

In the Cox PH model, HW with a high serological response presented a HR for BI of 0.27 [95% CI 0.11–0.66], when compared to HW with a medium-level serological response. Moreover, the stratified log-rank test for equality of survivor functions showed a significant difference between the three levels of serological response (*P* = 0.016). The test of the PH assumption based on Schoenfeld residuals revealed no evidence that the PH assumption has been violated (*P* = 0.918). A borderline significant HR was observed for female sex (HR = 0.59, 95% CI 0.34–1.01). Further details are presented in Table 3.

**Table 3:** Cox proportional hazards survival analysis of the pre-Omicron period

	HR	95% CI	*P* value
Sex (Reference: male)			
Female	0.59	0.34–1.01	0.056
Age (Reference: 18–29)			
30–39	0.91	0.34–2.49	0.859
40–49	1.06	0.44–2.50	0.911
50–59	0.94	0.36–2.44	0.902
≥ 60	0.53	0.17–1.65	0.272
Job title (Reference: physicians)			
Nurses	1.18	0.59–2.35	0.644
Technicians	0.35	0.09–1.32	0.120
Administrative	0.32	0.07–1.49	0.147
Other HCWs	0.61	0.23–1.61	0.321
Previous infections (Reference: no previous infections)			
Before 1st vaccine dose	1.22	0.20–6.19	0.894
After 1st vaccine dose			
Serological response (Reference: medium)			
High	0.27	0.11–0.66	0.004
Low	1.03	0.42–2.52	0.947

In the Phase 2 analysis, we found evidence of reduced risk of COVID-19 infection in three job titles when compared to physicians: nurses [HR = 0.84, 95% CI 0.74–0.95], administrative workers [HR = 0.72, 95% CI 0.60–0.86] and other working categories of HW [HR = 0.78, 95% CI 0.67–0.90]. A previous infection after the first vaccine dose entailed as well a reduced risk of COVID-19 infection, as compared to HW who had no previous infections [HR = 0.76, 95% CI 0.59–0.97]. Results of the Cox PH model also showed a strong relationship between a high-level serological response and a decreasing risk of COVID-19 infection, when compared to HW with medium-level serological response [HR = 0.76, 95% CI 0.62–0.93]. The KM survival curves related to the three different levels of serological response (Fig. 2) showed not only that HW with higher antibody levels (blue curve) presented higher survival probability at each time than HW, who had a medium-level serological response (green curve), but also that HW with lower antibody levels (red curve) presented lower survival probability at each time than HW who had a medium-level serological response (green curve).

Moreover, the stratified log-rank test for equality of survivor functions showed a significant difference between the three different levels of serological response (*P* = 0.041), as well as between the five age groups (*P* = 0.002) and the five job titles (*P* ≤ 0.001). The test of the PH assumption based on Schoenfeld residuals revealed no evidence that the PH assumption has been violated (*P* = 0.054). Further details are presented in Table 4.

**Table 4:** Cox proportional hazards survival analysis of the Omicron period

	HR	95% CI	*P* value
Sex (Reference: male)			
Female	0.98	0.89–1.09	0.766
30–39	1.04	0.88–1.21	0.669
40–49	0.91	0.78–1.05	0.208
50–59	0.89	0.77–1.03	0.117
≥ 60	0.86	0.71–1.04	0.119
Job title (Reference: physicians)			
Nurses	0.84	0.74–0.95	0.008
Technicians	0.85	0.72–1.00	0.063
Administrative	0.72	0.60–0.86	0.000
Other HCWs	0.78	0.67–0.90	0.001
Previous infections (Reference: no previous infections)			
Before 1st vaccine dose	1.03	0.85–1-25	0.739
After 1st vaccine dose	0.76	0.59–0.97	0.025
Serological response (Reference: medium)			
High	0.76	0.62–0.93	0.009
Low	1.03	0.91–1.18	0.621

## Discussion

The cumulative incidence of BI among vaccinated HW belonging to the ORCHESTRA Project was significantly higher during the spread of the OV, as compared to previous VOC periods (0.33% vs 20%). These results are in line with data on the general population, confirming that OV has increased contagiousness even among individuals who were vaccinated with a booster dose [17]. Furthermore, the same trend was shown in the study of Asamoah-Boaheng et al., which investigated the incidence of SARS-CoV-2 infection among 1000 paramedics in Canada. Indeed, they found a BI incidence of 1.5% and 13%, before and after the Omicron era, respectively [18].

The reduced effectiveness of vaccines against the new variants is due to the occurrence of mutations at the level of the surface protein S, the protein toward which vaccines stimulate the humoral response [19]. As a consequence, a reduction in protection of anti-S IgG levels toward infection risk can be expected.

Indeed, our study reported that, during POV, HW with a serological level of anti-S IgG above the 75th percentile had a 73% reduced risk of BI than those who had an antibody titer between the 25th and 75th percentile. On the other hand, low responders (below the 25th percentile) did not have a significantly increased risk. Similar results were found both in the general and HW populations. Aldridge et al. evaluated the effect of 1 Log unit increase on BI risk among 9244 individuals of the Virus watch Cohort. They found a HR of 0.85 and reported a lower risk of BI in subjects with an anti-S IgG level above the 75th percentile if compared to subjects who had an antibody level below the 25th, by day 20 of follow-up [20]. Seekircher et al. analyzed the protective role of anti-S IgG in the Austrian population. During the 6-month follow-up, they reported a diminished risk of BI in individuals with higher levels of anti-S IgG. In particular, having twice the immunological parameter was related to a HR of 0.72 [21]. An inverted correlation between anti-S RBD IgG levels and risk of BI was found also by Smoot et al., in a study that involved 2139 nursing home residents and staff during the Delta surge [22]. However, not all studies have detected a correlation between antibodies and the risk of BI. Indeed, Yang et al. investigated this aspect among 551 HW who underwent anti-S1 RBD titration from March to October 2021. The antibody geometric mean titer did not differ between HW who subsequently had a BI and those who were not infected. On the other hand, authors reported that only 1 out of 57 HW had a BI less than 10 weeks after the full vaccination. Almost all of them, therefore, occurred after this period, when the antibody titer had decreased by 55%. These data seem to suggest that the onset of BI was possible only after a significant reduction in the antibody level [23].

Considering the OV period, our survival analysis revealed that a high antibody level (above the 75th percentile) was protective against BI after the booster dose when compared to a medium response (HR = 0.76). Again, the lowest titer was not correlated with a higher risk. Several studies showed the same effect. Indeed, Asamoah-Boaheng et al. found a reduction in the risk of BI of 20% among paramedics [18]. The study of Mohlendick et al. involved 1391 boostered HW belonging to the University Hospital Essen. The results demonstrated a twofold increased risk of BI among HW with an anti-S1 RBD titer lower than 2816.0 BAU/ml when compared to HW who had an antibody level above this cut-off (OR = 2.12; CI 1.24–3.58) during the period between November 2021 and March 2022 [24]. Barda et al. used antibody levels before the 4th dose administration and observed BI incidence while OV was prevalent in Israel, up to 6 months after the second booster dose. Among the 1098 HW involved, an anti-S IgG level > 700 BAU/mL was related to a 35% BI risk reduction. Furthermore, a tenfold increase in IgG levels decreased the risk of BI by more than 50% [25]. Gilboa et al. investigated if a higher peak of anti-S RBD against SARS-CoV-2 after booster dose was protective against infection in a Cohort of 2865 naïve HW. They found that infected HW during the OV period had a lower peak than those not infected (2659 BAU/mL vs 3107 BAU/mL; ratio of means = 0.86) [26]. A previous study, carried out in the framework of the ORCHESTRA project, confirmed this effect. Indeed, they reported that a tenfold increase of anti-S IgG led to an OR of the risk of BI equal to 0.71. In addition, HW with an anti-S titer above 2000 BAU had an OR of 0.52 and 0.34 when compared to HW with a titer lower than 500 and 1024, respectively. On the other hand, the antibody level was not related to the virus infectivity [27].

As in the POV period, some studies did not detect a protective effect of antibody titer. Indeed, among the 527 HW involved in the study of Dodge et al., although subjects who were infected by OV had lower anti-S IgG titers before positivity than negative ones, the difference was not statistically significant [28]. In the same way, even Santoro et al. reported that there was no correlation between anti-S RBD levels and OV infection in a similar sample (487 HW) [29].

Comparing the effect of antibody titer in the two periods, our data showed that while it still had a protective role in both periods, its effectiveness was significantly reduced after the onset of OV. Indeed, the risk reduction dropped from −73 to −24%. Asamoah-Boaheng et al. reported a decline from −35 to −20%, before and after the OV period [18]. The reduction in the protective effect was also reported by Smoot et al., who showed an inverse correlation only during the Delta variant period, while no correlation after the onset of the OV [22]. A previous study from one of the cohorts belonging to ORCHESTRA Project (Brescia), evaluated the effect of the SARS-CoV-2 anti-S antibody level on the risk of infection in 4824 HW. It revealed that the number of infections caused by the OV were noticeably higher (12 ×) than those caused by POV during a similar follow-up period (7–9 months). Furthermore, the number of SARS-CoV-2 infections among HW with higher serological response was not-neglectable (5.6% during a 6–8 months period) [30].

These findings supported the hypothesis that, rather than referring BI to the limited effectiveness of anti-SarS-CoV-2 vaccinations, the observation of new infection cases (BI), and even their increase during the phases of the pandemic following the first wave when vaccination was not yet available, can be referred to the occurrence of new variants including Omicron.

Such information is relevant, in order to appraise the development of the COVID-19 pandemic, both for the governmental institutions and the general public. In fact, the occurrence of new cases of infection in the vaccinated contributed to the poor trust of the public to vaccination campaigns.

These data seem to confirm that, although a higher anti-S titer is a predictor of the risk of BI even during VOC periods it cannot be used to exclude the risk of being infected by SARS-CoV-2 and, therefore, being infectious. Similarly, antibody levels in vaccinated HW is not likely to be a useful parameter to evaluate the timing for further booster dose. Therefore, it is a priority to investigate other possible indicators of protection that may have a stronger and longer relation with the risk of BI, such as cellular immunity.

Our study has some limitations.

First of all, subjects were classified as infected by POV or OV based only on the date of PCR positivity. Indeed, no laboratory data on strain type were available and some cases could therefore be misclassified. Furthermore, serological assays were performed with different methods among cohorts. To analyze the data jointly, antibody levels were normalized on a logarithmic scale, using a method already applied in previous studies of the ORCHESTRA project [11–14]. This prevents the interpretation of our results along a unique scale of anti-S level measurements. In most cohorts, the measurement of anti-S level was idiosyncratic rather than following a fixed schedule. Moreover, HW participating in the POV and OV periods were slightly different, regarding age and proportion of women (69.0% vs 73.3%), physicians (27.6% vs 21.8%), administrative staff (7.2% vs 10.3%) and, as expected, previously infected (0% vs 8.1%). However, multivariable survival analysis was adjusted for these variables to prevent/minimize confounding bias. Finally, clinical data were available only in a subset of HW, and the analysis of the correlation between anti-S levels and symptomatic or asymptomatic infections was therefore not performed.

Our study has also some strengths.

To the best of our knowledge, this study is the one exploring the correlation between antibodies and BI risk with the highest number of HW involved. Another strength is that our study is multicentric, including 11 different cohorts belonging to 4 European countries. In addition, our study investigated the effect of anti-S IgG on BI risk in two different periods with a follow-up of about 6 months, enabling to compare trends in different pandemic eras, in contrast to the majority of previous studies, that analyzed only one period.

## Conclusion

Our data confirmed that a higher anti-S level was protective against infection during the POV period, as well as during the Omicron surge, among a large population of vaccinated HW, belonging to different European Cohorts. However, the protection was not complete and waned significantly after the emergence of new variants; therefore, the anti-S titer is not suitable as the predictor of the timing for further booster dose administration. Studies on cellular immunity against SARS-CoV-2 are therefore needed to better understand the mechanisms and duration of protection vs the risk of BI.

### Supplementary Information

Below is the link to the electronic supplementary material.

Supplementary file1 (DOCX 359 KB)

## Data Availability

The datasets generated during the current study are not accessible to the public due to the inclusion of sensitive information necessitating compliance with data protection statutes and guidelines. Appropriate forms of data sharing can be arranged after a substantiated request to the last author.
