# Supplementary material for: Protective role of SARS-CoV-2 anti-S IgG against breakthrough infections among European healthcare workers during pre and post-Omicron surge—ORCHESTRA project
Source: Infection. 2024 Feb 7;52(4):1347–56. doi: 10.1007/s15010-024-02189-x (PMC11289150; doi:10.1007/s15010-024-02189-x)

**Supplementary material**

Figure S1 - Data selection for Phase 1 analysis
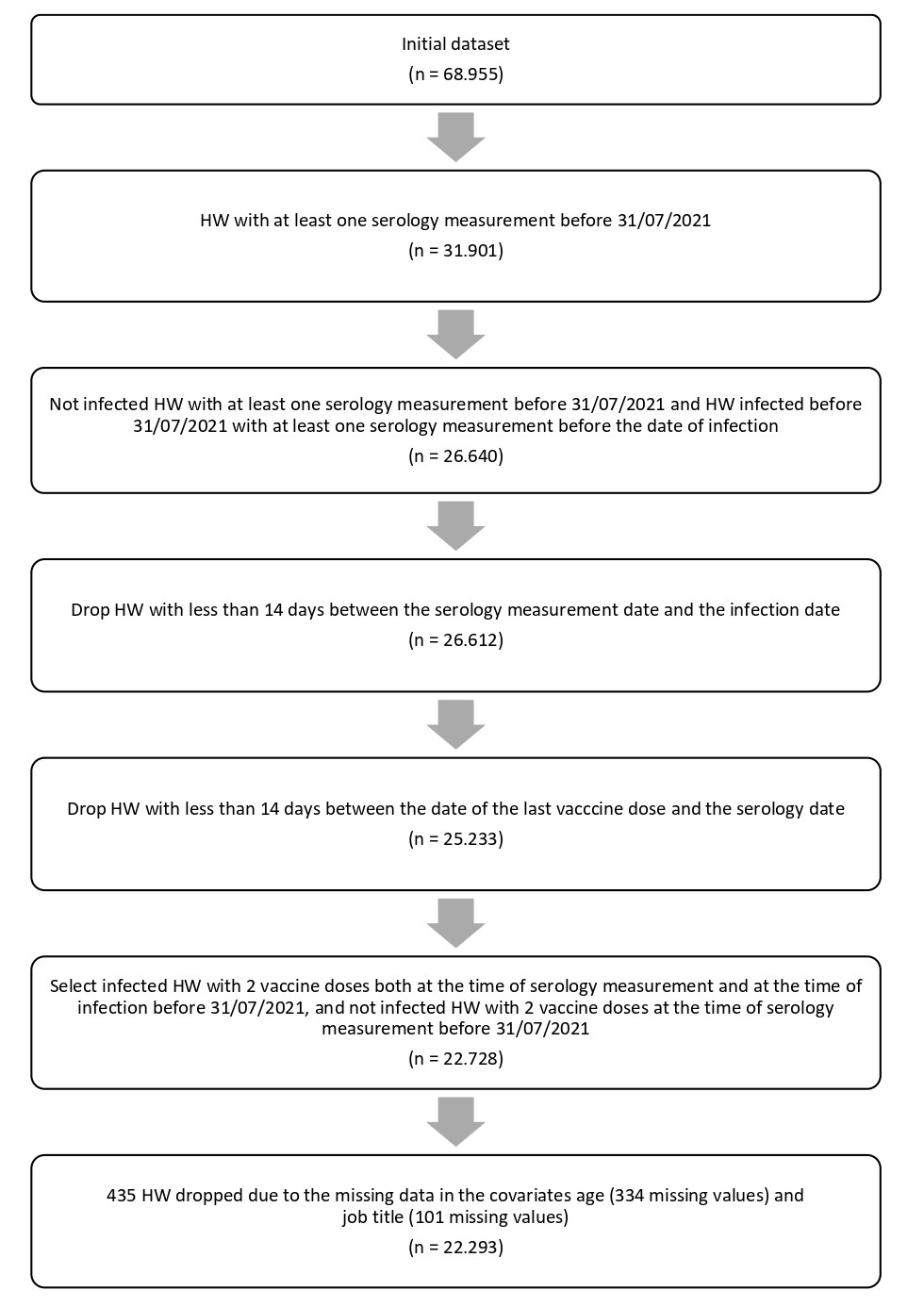


Figure S2 - Data selection for Phase 2 analysis


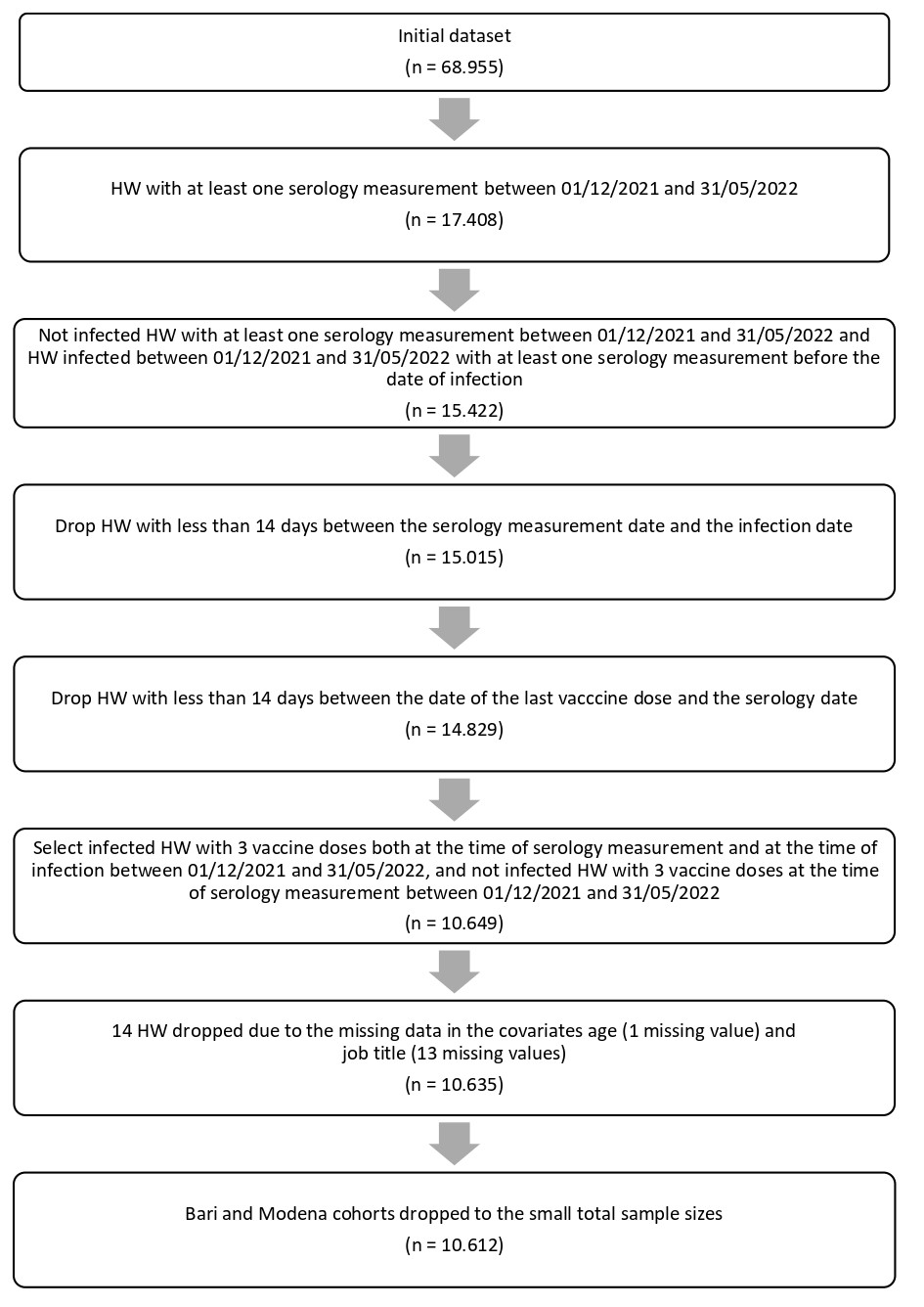

Supplement: Supplementary file 1 — Supplementary file1 (DOCX 359 KB) [file 15010_2024_2189_MOESM1_ESM.docx]
